# Compliant substratum modulates vinculin expression in focal adhesion plaques in skeletal cells

**DOI:** 10.1038/s41368-019-0052-3

**Published:** 2019-06-01

**Authors:** Chenchen Zhou, Qingxuan Wang, Demao Zhang, Linyi Cai, Wei Du, Jing Xie

**Affiliations:** State Key Laboratory of Oral Diseases, West China Hospital of Stomatology, Sichuan University, Chengdu, China

**Keywords:** Biological fluorescence, Molecular biophysics

## Abstract

The biophysical properties of the extracellular matrix (ECM) dictate tissue-specific cell behaviour. In the skeleton system, bone shows the potential to adapt its architecture and contexture to environmental rigidity via the bone remodelling process, which involves chondrocytes, osteoblasts, osteoclasts, osteocytes and even peripheral bone marrow-derived stem/stromal cells (BMSCs). In the current study, we generated stiff (~1 014 ± 56) kPa, Young’s modulus) and soft (~46 ± 11) kPa silicon-based elastomer polydimethylsiloxane (PDMS) substrates by mixing curing agent into oligomeric base at 1:5 and 1:45 ratios, respectively, and investigated the influence of substrate stiffness on the cell behaviours by characterizing cell spreading area, cell cytoskeleton and cell adhesion capacity. The results showed that the cell spreading areas of chondrocytes, osteoblasts, osteoclasts, osteocytes and BMSCs were all reduced in the soft substrate relative to those in the stiff substrate. F-actin staining confirmed that the cytoskeleton was also changed in the soft group compared to that in the stiff group. Vinculin in focal adhesion plaques was significantly decreased in response to soft substrate compared to stiff substrate. This study establishes the potential correlation between microenvironmental mechanics and the skeletal system, and the results regarding changes in cell spreading area, cytoskeleton and cell adhesion further indicate the important role of biomechanics in the cell-matrix interaction.

## Introduction

The physiological activities in the vertebrate body are regulated by a cascade of chemical signalling events. These events intertwine to guide cell-cell contacts and cell-matrix interactions and eventually modulate embryonic development,^1^ tissue remodelling,^2^ immune surveillance^3^ and other homeostatic processes.^4^ However, tissues and organs are always experiencing an ever-changing mechanical stimulus, such as blood pressure, or a specific physical microenvironment from the very soft gyri and sulci of the brain to the very hard rigid bone tissue.^5^ These physical forces, including fluid shear stress in blood flow, pressure in the trachea and bronchus and even local extracellular matrix (ECM) stiffness, can influence tissues not only by directing changes in cell shape but also by affecting a variety of cellular processes,^6^ among which the most important one might be cell adhesion.^7^

Cell-matrix adhesion is distributed on the cell surface and modulates the cell interaction with its peripheral ECM.^8^ Adhesion is important due to its vital role in modulating cellular functions, such as proliferation, division, migration, differentiation and autophagy.^9^ The primary adhesions can either be quickly disassembled or progressively stabilized to form fibrillar adhesions through changing the shapes and molecular components in the adhesion complexes.^10^ Vinculin is a major regulator of cell adhesion and attaches to the cell surface by interaction with the specific phospholipids in adhesion complexes.^11^ This molecule has emerged as an early and essential characteristic of nascent cell-matrix adhesion and acts as a scaffold to sustain many actin-organizing proteins.^12^ In mature focal adhesions (FAs), vinculin also plays a key role as a ‘molecular clutch' that modulates the transmission of mechanical force from the membrane-bound integrins to cytoplasmic F-actin.^13^ In the transgenic mouse model, vinculin-deficient mice are small (embryos at E9.5 are approximately a third smaller than normal embryos) and die at day E10.5, with major defects in brain and heart development.^14,15^ Mouse embryonic fibroblasts isolated from vinculin knockout embryos at E9.5 spread less, have smaller FAs and show decreased adhesion strength to fibronectin, laminin, vitronectin and collagen.^15^

ECM stiffness has been identified as an important contributor in malignant transformation and tumour metastasis by activating vinculin at the invasive border of tumours.^16^ Here, we aimed to explore stiffness-mediated vinculin changes in skeletal system cells. Using silicon-based elastomer polydimethylsiloxane (PDMS), we fabricated substrates with different stiffnesses and aimed to reveal the vinculin changes in chondrocytes, osteoblasts, osteoclasts, osteocytes and peripheral bone marrow-derived stem/stromal cells (BMSCs) in response to PDMS substrates with varied stiffnesses.

## Results

### Compliant substrate reduces vinculin expression in chondrocytes

**Fig. 1:**
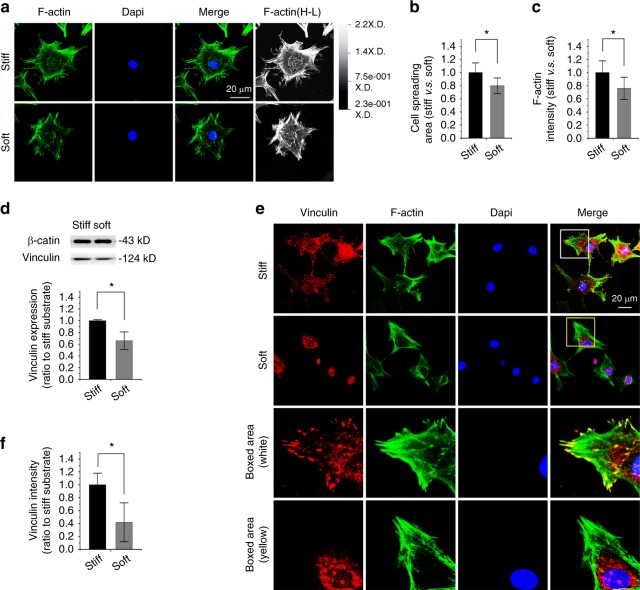
Substrate elasticity alters vinculin expression in chondrocytes. **a** Representative CLSM images showing cytoskeletal changes by characterizing F-actin in chondrocytes in response to substrates with different stiffnesses (*n* = 4). Changes of F-actin in chondrocytes were also presented in the High-Low model in CLSM. **b** Histogram showing the change in cell spreading area of chondrocytes in response to substrates with different stiffnesses. Statistical data are based on more than 100 cells in each group in four independent experiments (*n* > 100). *P < 0.05. **c** Histogram showing the change in F-actin by immunofluorescence intensity in chondrocytes in response to substrates with different stiffnesses. Statistical data are based on more than 100 cells in each group in four independent experiments (*n* = 4). *P < 0.05. **d** Western blotting showing the change of vinculin in chondrocytes in response to substrates with different stiffnesses. Quantification was then performed to confirm the changes (*n* = 3). *P < 0.05. **e** Representative CLSM images showing the change in vinculin by immunofluorescence staining in chondrocytes in response to substrates with different stiffnesses. **f** Histogram showing the change in vinculin in (**e**) chondrocytes in response to substrates with different stiffnesses. Statistical data are based on more than 80 cells in each group in four independent experiments (*n* = 4). *P < 0.0

Based on our previous reports about the influence of varied PDMS substrates on myocardial cells^17^ and adipose-derived stromal cells,^18^ in this study, we detected the potential impact of elastic PDMS substrate as a vital physical stimulus on cell adhesion in skeletal cells by characterizing vinculin, a membrane-cytoskeletal protein in focal adhesion plaques and in charge of linking integrin adhesion molecules to the actin cytoskeleton.^19^ In primary mouse chondrocytes, we first found changes in the cytoskeleton by F-actin staining in chondrocytes in response to different substrate stiffnesses (Fig. 1a). The cell spreading area was reduced to approximately 80% in soft substrate relative to that in stiff substrate (Fig. 1b). The quantification of fluorescent intensity in F-actin further showed that the smaller cells in soft substrate showed weaker fluorescent intensity (Fig. 1c). We then examined vinculin expression in chondrocytes and found it was reduced in soft substrate relative to that in stiff substrate by Western blotting (Fig. 1d). The fluorescent staining of vinculin further showed its distribution changes in chondrocytes in response to different substrate stiffnesses (Fig. 1e). In the stiff substrate, its distribution was shown to be spot-like along the border of the cell membrane, but in the soft substrate, the distribution was confined to the region around the nucleus. The quantification of fluorescent intensity in vinculin further showed a reduced intensity in chondrocytes in response to soft substrate compared to stiff substrate (Fig. 1f).

### Compliant substrate reduces vinculin expression in osteoblasts

**Fig. 2:**
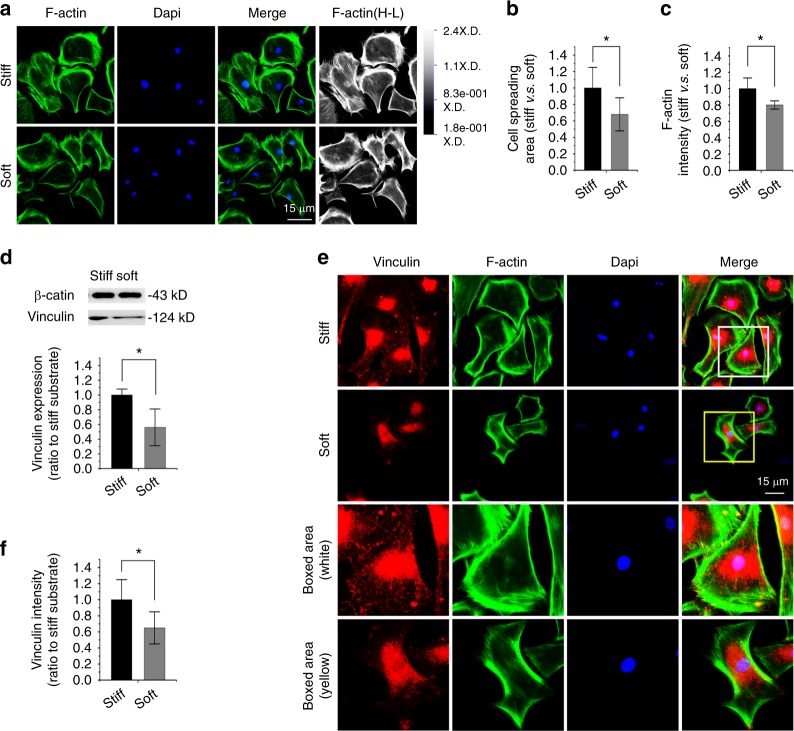
Substrate elasticity alters vinculin expression in osteoblasts. **a** Representative CLSM images showing cytoskeletal changes by characterizing F-actin in osteoblasts in response to substrates with different stiffnesses (*n* = 3). Changes of F-actin in osteoblasts are also presented in the High-Low model in CLSM. **b** Histogram showing the change in the cell spreading area of osteoblasts in response to substrates with different stiffnesses. Statistical data are based on more than 110 cells in each group in three independent experiments (*n* = 3). *P < 0.05. **c** Histogram showing the change of F-actin by immunofluorescence intensity in osteoblasts in response to substrates with different stiffnesses. Statistical data are based on more than 110 cells in each group in three independent experiments (*n* = 3). *P < 0.05. **d** Western blotting showing the change of vinculin in osteoblasts in response to substrates with different stiffnesses. Quantification was then performed to confirm the changes (*n* = 3). *P < 0.05. **e** Representative CLSM images showing the change in vinculin by immunofluorescence staining in osteoblasts in response to substrates with different stiffnesses. **f** Histogram showing the change of vinculin in (**e**) osteoblasts in response to substrates with different stiffnesses. Statistical data are based on more than 100 cells in each group in three independent experiments (*n* = 3). *P < 0.05

We then investigated the influence of substrate stiffness on primary osteoblasts. We showed cytoskeletal changes by characterizing F-actin in osteoblasts in response to different substrate stiffnesses (Fig. 2a). The cell spreading area in the soft substrate was reduced relative to that in the stiff substrate (Fig. 2b). The quantification of fluorescent intensity in F-actin in response to soft substrate showed a reduced intensity in osteoblasts (Fig. 2c), which was inferior to that in chondrocytes (Fig. 1c). The next investigation by Western blotting of vinculin indicated that its expression in osteoblasts was reduced in response to soft substrate relative to stiff substrate (Fig. 2d). Fluorescence staining of vinculin showed changes in the distribution of osteoblasts in response to substrates with different stiffnesses (Fig. 2e). In the stiff substrate, vinculin was not only distributed near the nucleus but also dispersed widely in the cytoplasm adhered to the PDMS substrate, but in the soft substrate, the distribution of vinculin was largely reduced in the cytoplasmic region and confined to the nuclear region. The quantification of fluorescence intensity in vinculin further showed a reduced intensity in osteoblasts in response to soft substrate compared to stiff substrate (Fig. 2f).

### Compliant substrate reduces vinculin expression in osteoclasts

**Fig. 3:**
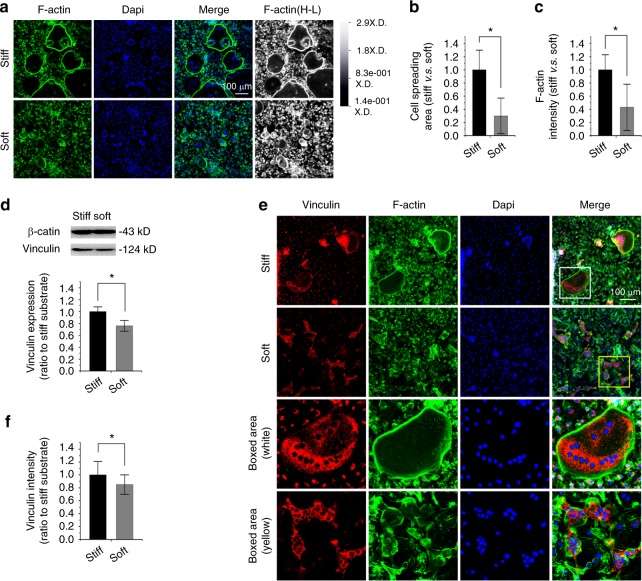
Substrate elasticity alters vinculin expression in osteoclasts. **a** Representative CLSM images showing cytoskeletal changes by characterizing F-actin in osteoclasts in response to substrates with different stiffnesses (*n* = 5). Changes in F-actin in osteoclasts are also presented in the High-Low model in CLSM. **b** Histogram showing the change in cell spreading area of osteoclasts in response to substrates with different stiffnesses. Statistical data are based on more than 90 cells in each group in five independent experiments (*n* = 5). *P < 0.05. **c** Histogram showing the change of F-actin by immunofluorescence intensity in osteoclasts in response to substrates with different stiffnesses. Statistical data are based on more than 90 cells in each group in five independent experiments (*n* = 5). *P < 0.05. **d** Western blotting showing the change in vinculin in osteoclasts in response to substrates with different stiffnesses. Quantification was then performed to confirm the changes (*n* = 3). *P < 0.05. **e** Representative CLSM images showing the change in vinculin by immunofluorescence staining in osteoclasts in response to substrates with different stiffnesses. **f** Histogram showing the change of vinculin in (**e**) osteoclasts in response to substrates with different stiffnesses. Statistical data are based on more than 90 cells in each group in five independent experiments (*n* = 5). *P < 0.05

We next investigated the influence of substrate stiffness on osteoclasts, which was shown to play a contrary role to osteoblasts in bone development and remodelling.^20,21^ First, to obtain many bone marrow macrophages (BMMs) from femurs, we induced primary bone marrow cells with MCSF at 40 ng·mL^−1^ for 24 h. We then collected the suspended BMMs and seeded them onto PDMS substrates after cell counting. Then, the cells were maintained in the induction culture media with 20 ng·ml^−1^ M-CSF and 20 ng·mL^−1^ RANKL. The suspended cells completed the attachment onto the PDMS substrates and began osteoclast differentiation within two days. After approximately 4 days, large fused osteoclasts could be observed. Interestingly, the soft substrate could not form large osteoclasts as did the stiff substrate after being induced for the same time and with the same concentration of macrophage colony-stimulating factor (MCSF, 20 ng·mL^−1^) and Receptor Activator of Nuclear Factor-κ B Ligand (RANKL, 20 ng·mL^−1^). This result was confirmed by F-actin staining (Fig. 3a). In the stiff substrate, osteoclasts could fuse into larger particles with increased nuclei accumulation. The actin rings were shown to be brighter and larger, and they directly indicated the size of a fused osteoclast. In the soft substrate, there were many smaller but fused osteoclasts with fewer nuclei accumulated. We calculated the size of fused osteoclasts and found that the size was reduced to approximately 30% in soft substrate compared to stiff substrate (Fig. 3b). The total fluorescence intensity of the actin ring was correspondingly reduced to approximately 40% (Fig. 3c). We next explored vinculin expression in osteoclasts in response to substrate stiffness. The total vinculin expression was reduced to 76% in soft substrate relative to stiff substrate by Western blotting (Fig. 3d). By IF staining, we found that the distribution in osteoclasts was mainly gathered around the clustered nuclei (Fig. 3e). The vinculin expression was significantly reduced in soft substrate relative to that in stiff substrate. The quantification of fluorescence intensity in vinculin further showed a reduced intensity in osteoclasts in response to soft substrate compared to stiff substrate (Fig. 3f).

### Compliant substrate reduces vinculin expression in osteocytes

**Fig. 4:**
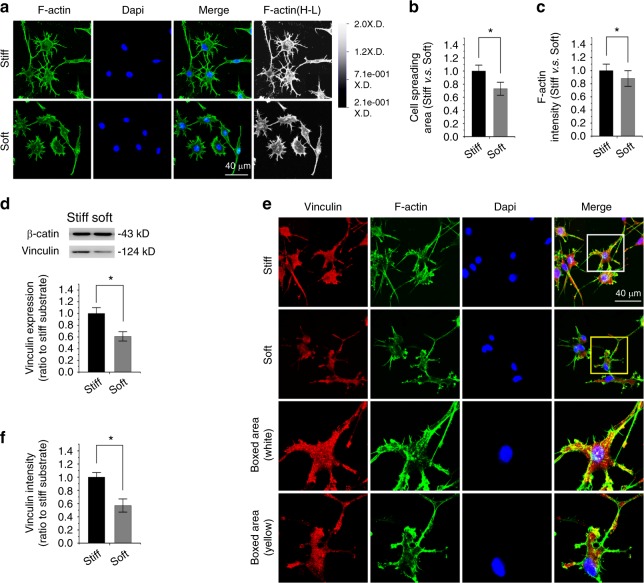
Substrate elasticity alters vinculin expression in osteocytes. **a** Representative CLSM images showing cytoskeletal changes by characterizing F-actin in osteocytes in response to substrates with different stiffness (*n* = 4). Changes in F-actin in osteocytes are also presented in the High-Low model in CLSM. **b** Histogram showing the change in cell spreading area of osteocytes in response to substrates with different stiffnesses. Statistical data are based on more than 130 cells in each group in four independent experiments (*n* = 4). *P < 0.05. **c** Histogram showing the change of F-actin by immunofluorescence intensity in osteocytes in response to substrates with different stiffnesses. Statistical data are based on more than 130 cells in each group in four independent experiments (*n* = 4). *P < 0.05. **d** Western blotting showing the change of vinculin in osteocytes in response to substrates with different stiffnesses. Quantification was then performed to confirm the changes (*n* = 3). *P < 0.05. **e** Representative CLSM images showing the change in vinculin by immunofluorescence staining in osteocytes in response to substrates with different stiffnesses. **f** Histogram showing the change in vinculin in (**e**) osteocytes in response to substrates with different stiffnesses. Statistical data are based on more than 100 cells in each group in four independent experiments (*n* = 4). *P < 0.05

We used a well-recognized cell line, MLO-Y4, to investigate the influence of substrate stiffness on the osteocytes. By F-actin staining, we found a cytoskeletal change in osteocytes in response to substrate stiffnesses (Fig. 4a). The cell dendritic process was weakened in soft substrate relative to that in stiff substrate. We calculated the size of the cell spreading area and found that it was reduced to approximately 73% in soft substrate compared to stiff substrate (Fig. 4b). The total fluorescence intensity of F-actin was correspondingly reduced to approximately 88% (Fig. 4c). We performed Western blotting of vinculin (Fig. 4d) and found that its expression was reduced to 61% in soft substrate compared to that in the stiff substrate. IF staining further confirmed that the expression of vinculin in the soft substrate was reduced compared to the stiff substrate (Fig. 4e). The quantification of fluorescence intensity in vinculin further showed the reduced intensity in osteocytes in response to soft substrate compared to stiff substrate (Fig. 4f).

### Compliant substrate reduces vinculin expression in peripheral bone marrow-derived stem/stromal cells (BMSCs)

**Fig. 5:**
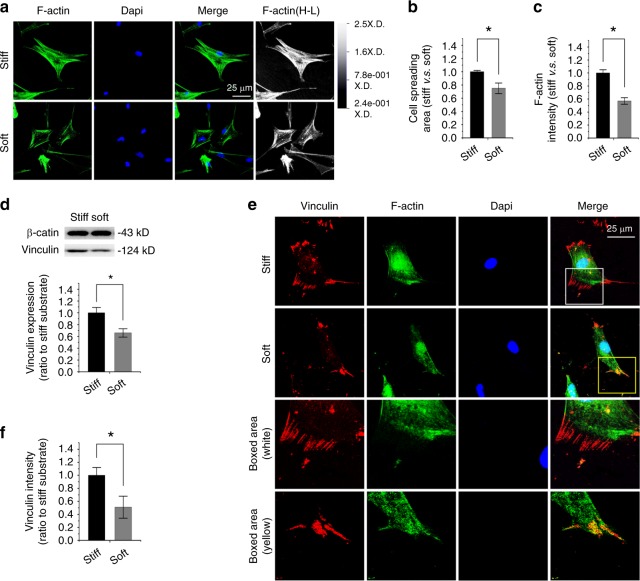
Substrate elasticity alters vinculin expression in bone marrow stromal cells (BMSCs). **a** Representative CLSM images showing cytoskeletal changes by characterizing F-actin in BMSCs in response to substrates with different stiffnesses (*n* = 3). Changes of F-actin in BMSCs are also presented in the High-Low model in CLSM. **b** Histogram showing the change in cell spreading area of BMSCs in response to substrates with different stiffnesses. Statistical data are based on more than 70 cells in each group in three independent experiments (*n* = 3). *P < 0.05. **c** Histogram showing the change in F-actin by immunofluorescence intensity in BMSCs in response to substrates with different stiffnesses. Statistical data are based on more than 70 cells in each group in three independent experiments (*n* = 3). *P < 0.05. **d** Western blotting showing the change in vinculin in BMSCs in response to substrates with different stiffnesses. Quantification was then performed to confirm the changes (*n* = 3). *P < 0.05. **e** Representative CLSM images showing the change of vinculin by immunofluorescence staining in BMSCs in response to substrates with different stiffnesses. **f** Histogram showing the change in vinculin in (**e**) BMSCs in response to substrates with different stiffnesses. Statistical data are based on more than 90 cells in each group in four independent experiments (*n* = 4). *P < 0.05

We finally investigated the influence of substrate stiffness on BMSCs isolated from long bone marrow (mouse femur). By F-actin staining, we found cytoskeletal changes in BMSCs in response to different substrate stiffnesses (Fig. 5a). F-actin was shown to be brighter and displayed a bundle-like distribution in response to stiff substrate, while on the soft substrate, it seemed to be weaker and more scattered. By calculating the cell spreading area, we found that it was reduced to 75% in soft substrate compared to that in stiff substrate (Fig. 5b). The fluorescence intensity of F-actin correspondingly decreased to approximately 57% in soft substrate relative to that in stiff substrate (Fig. 5c). We then investigated the expression of vinculin in BMSCs in response to different substrate stiffnesses. By Western blotting, we found that the expression of vinculin was decreased to approximately 66% in soft substrate relative to that in stiff substrate (Fig. 5d). By immunofluorescence, we confirmed the decrease of vinculin in the soft substrate and found its distribution along the outside border of the cell membrane (Fig. 5e). The bundle-like distribution of vinculin was completely perpendicular to the direction of the cell membrane. Although this trend in vinculin occurred in both soft and stiff substrates, the quantification of fluorescence intensity indicated a decrease in BMSCs in response to soft substrate compared to stiff substrate (Fig. 5f).

## Discussion

Cell-matrix adhesion is considered to be an essential factor for physiological function in all kinds of cells embedded in multicellular organisms. The cells need to mediate their behaviours in response to external stimuli by changing their cell-matrix linkages.^22,23^ These dynamic and homeostatic changes in cell-matrix adhesion show their potential for cell motility, tissue development, ECM remodelling, wound healing and immune surveillance and many pathological processes, such as myocardial fibrosis,^17^ hepatocirrhosis^24^ and osteoporosis.^25^ In this process of adhesion maturation, mechanical forces, accompanied by chemical signals, play a key role.^17,18^ In this study, to mimic different stiffnesses generated by ECM in tissues, we fabricated substrates with different stiffnesses by controlling the ratios of curing agent and oligomeric base in PDMS materials. We seeded the skeletal cells, e.g., chondrocytes, osteoblasts, osteoclasts, osteocytes and peripheral BMSCs, onto the PDMS substrates with different stiffnesses and elaborated the changes of cell spreading areas, cytoskeletons and the focal adhesion protein vinculin. This study provided an intuitive result about the role of interface stiffness on the cells in skeletal systems.

Polydimethylsiloxane (PDMS) has been widely used in biomaterial studies due to its advantages, such as biocompatibility, optical characteristics and elastic plasticity.^18,26^ By using PDMS biomaterials, many biological processes have been elucidated, for example, the mechanism of myocardial fibrosis,^17^ the geometric guidance for cell life and death,^27^ the differentiation of adipose-derived stromal cells (ASCs) in response to ECM stiffness,^18^ small molecule changes in the subcellular fraction by mechanical forces^28^ and even in single cell investigations by devices made of PDMS.^29^ The PDMS provides an in vitro research model for the interaction between cells and matrix in vivo. For adult cells in the body, Gilbert et al.^30^ revealed that cardiosphere-derived cells could express more endothelial markers and increase cell survival in stiffened cues.^30^ For stem cells, Engler et al.^31^ first elucidated that bone marrow mesenchymal stem cells (BMSCs) could direct cell commitment into adipogenic, myogenic, and osteogenic lineages in response to low, intermediate, and high stiffness, respectively. In our study, we showed that different stiffnesses could modulate the cell spreading area, cytoskeleton, and a focal adhesion protein, vinculin, in chondrocytes, osteoblasts, osteoclasts, osteocytes and BMSCs. We showed that soft PDMS substrate ((0.046 ± 0.020) MPa) could reduce the cell spreading area and cytoskeleton distribution and the expression of vinculin in comparison to the stiff PDMS substrate ((1.014 ± 0.150) MPa). These results are significant because they provide the landscape in stiffened substrate-mediated changes in skeletal cells and will guide us to explore the deeper mechanisms underlying these phenotypes in our future work.

In regard to the interaction between cells and the interface, three major factors must be involved: geometry, hydrophilicity, and stiffness.^18^ For the surface geometry, it was demonstrated that stem cell osteogenesis was enhanced when the cells were confined to shapes with increased cytoskeletal tension. The dimension can also promote rat BMSC proliferation on more unevenly dimensioned substrates with the same stiffness and topography.^32^ Different surface geometries also promoted cell morphology changes and cell proliferation.^33^ Surface roughness could also influence cell functions, such as cell migration, cell proliferation and differentiation.^34^ For hydrophilicity, Sun et al.^35^ systematically summarized and illustrated the biological processes influenced by the hydrophilicity from structural biointerface materials and smart biointerface materials. Menon et al.^36^ found that the migration of human bone marrow-derived mesenchymal stem cells (hBMSCs) was greatly enhanced when cells were seeded on the PDMS substrate with intermediate levels (1:20) and that the proliferation was increased when the cells were seeded on the PDMS substrate with a higher stiffness. In the current study, we fabricated PDMS substrates with the same geometry, especially the same surface roughness, but with different stiffnesses. Using this method, we only need to consider the influence of stiffness on the cell behaviours, avoiding the impact of surface roughness.^18^ Consequently, the results in the current study could reflect an accurate phenotype of skeletal cells in response to stiffness.

**Fig. 6:**
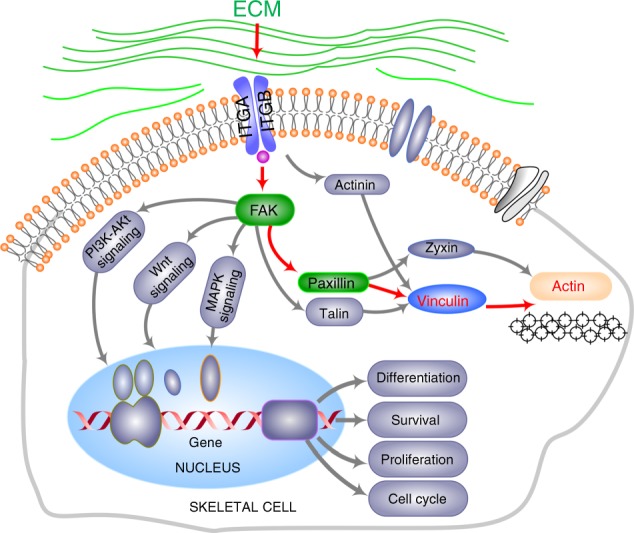
Schematic diagram elucidating the role of vinculin in the axis of ECM receptor—Focal adhesion—Cytoskeleton pathways. The arrows in red denote the modulating path in vinculin and F-actin cytoskeleton. The role of FAK and paxillin in green has been elucidated in our previous data.^19,34^ The grey parts are involved in the vinculin-cytoskeleton rearrangements but not in our study

Cells can sense environmental mechanical stiffness through active mechanosensing. The processes of this active mechanosensing and mechanotransduction of ECM mechanics into intracellular signals are mainly mediated by transmembrane receptors known as integrins (Fig. 6).^37^ Integrins include 18 α- and 8 β-subunits and can form at least 24 distinct αβ combinations.^38^ They serve as a ‘bridge' to connect the ECM and the cytoskeleton.^8^ On one hand, their ectodomains bind to ECM glycoproteins, collagens, other cellular receptors (e.g., vascular cell adhesion molecule-1) and the intercellular cell adhesion molecule family (e.g., ICAM-1 and ICAM-3);^39^ on the other hand, their cytoplasmic tails interact with adaptor molecules, such as talin in the focal adhesion plaque (FA).^40^ Competitive cytoplasmic binding with integrins regulates mechanical intracellular signal transduction. In the focal adhesion pathway, focal adhesion kinase (FAK), a cytoplasmic tyrosine kinase, is considered to interact with integrins through a FERM domain,^41^ which is found in the talin head and interacts with the integrin β leg or interacts indirectly through vinculin or paxillin.^42^ The changes in FA molecules further trigger the activation of cytoplasmic signal pathways and then promote the nuclear translocation of their downstream proteins and finally modulate gene expression.^17,18,43^ In osteocytes, we showed that osteocytes sense substrate stiffness through integrin αvβ3, which is predominant in mammalian osteocytes, and achieve substrate attachment and form FA complexes. Integrin αvβ3 then activates FAK signalling by direct protein binding. FAK, together with its adapter, paxillin, further triggers cytoplasmic β-catenin signalling and promotes its nuclear translocation.^19^ In dental papilla cells (DPCs), we found that paxillin interacts with both ectoplasmic fibronectin and cytoplasmic β-catenin signalling by direct binding and plays a vital role in DPC mineralization.^34^ In chondrocytes, we have detected the importance of the RhoA/ROCK pathway in the maintenance of the chondrocyte phenotype,^43^ but the specific biomechanical control mechanism is deficient. In addition, further elucidation of the modulation process of mechanosensing and mechanotransduction in other cells, such as osteoblasts and osteoclasts, in the skeletal system is needed.

Notably, vinculin, as a major regulator in the cell focal adhesion pathway (Fig. 6), is important in transmitting mechanical forces and orchestrating mechanical signalling events.^13–15^ Impairment in vinculin resulted in altered cell adhesion, contractility, motility and growth, all of which are important processes in development and physiological activity.^44^ A previous report has shown that vinculin has no enzymatic activity, but it can bind to actin, stimulating actin polymerization and recruiting actin remodelling proteins.^4^ We demonstrated the expression changes of vinculin in chondrocytes, osteoblasts, osteoclasts, osteocytes and BMSCs in response to substrates with different stiffnesses. These cells seeded onto the stiff substrate display the cell morphologies and vinculin expression, especially in chondrocytes, osteoblasts and BMSCs, close to those in the petri dishes, of which the stiffnesses were in the gigapascal range (GPa) and far stiffer than those in our current study and in the stiffest living tissue, such as bone. The cells on the soft substrate showed smaller cell spreading areas and expressed less vinculin. Most importantly, vinculin expression was not distributed along with actin distribution but was concentrated around the nuclear region, especially in chondrocytes, osteoblasts and osteocytes. These changes in vinculin accompanied with the decrease in substrate stiffness were all consistent with previous reports in transgenic mouse models.^13–15^ The change in vinculin provides a cue for us to improve the advantages of biomaterials in future work.

The current study has some limitations. First, although we provide the full landscape on substrate-regulated vinculin in skeletal cells, the specific biological mechanism is further needed to increase the understanding of cell-matrix interactions. Second, we used primary isolated chondrocytes, osteoblasts, osteoclasts and peripheral BMSCs, but the MLO-Y4 cell line represented mature osteocytes. The cell line can mimic but not reflect the actual morphological and biological changes of osteocytes, which might weaken the conclusion in the osteocyte part. Third, in the skeletal system, the cells, such as chondrocytes, osteoblasts, osteoclasts, osteocytes and peripheral BMSCs, are located in different mechanical environments and are physiologically stimulated by mechanics with different magnitudes and different types. The results shown in the study are based on the same mechanical stimulation and cannot represent the real mechanical stimulus occurring in each cell. These findings only provide us with a clue regarding tissue engineering and pathology.

In summary, this study described the influence of substrate stiffness on changes in the cell cytoskeleton and cell adhesion in skeletal cells by characterizing F-actin and vinculin expression. Based on the PDMS, the stiff and soft substrates were generated by mixing the curing agent into the oligomeric base at 1:5 and 1:45 ratios, respectively. The skeletal cells, e.g., chondrocytes, osteoblasts, osteoclasts, osteocytes and BMSCs, were seeded onto the stiff and soft substrates for 48 h and prepared for immunofluorescence detection. F-actin staining showed that cell spreading areas and cytoskeletons of skeletal cells in soft substrate were reduced relative to those in stiff substrate, and vinculin staining further indicated that the cell adhesion in soft substrate was significantly decreased compared to that in the stiff substrate. This study establishes the correlation between microenvironmental mechanics and skeletal cells, and the results regarding changes in the cytoskeleton and cell adhesion further emphasize the importance of biomechanics in the microenvironment on the cell-matrix interaction.

## Materials and methods

### Animals

The animal materials used for this study were obtained according to ethical principles, and all protocols were approved by the Institutional Review Board (IRB) of Sichuan University (IRB at the West China Hospital of Stomatology, No. WCHSIRB-D-2017-029). Newborn and one-month-old C57BL mice were obtained from the Experimental Animal Center of Sichuan University and housed in pathogen-free facilities under a 12-h light and 12-h dark cycle. Newborns provided primary chondrocytes and osteoblasts, and one-month-old mice provided long bone marrow for peripheral bone marrow-derived stem/stromal cells (BMSCs).

### PDMS substrate preparation

The preparation of PDMS substrates was previously described.^17,18^ In brief, PDMS substrates were fabricated by mixing curing agent (Sylgard 184) and oligomeric base (Corning, NY, USA). The ratios of 1:5 and 1:45 were used to achieve the stiff substrate with (~1.014 ± 0.150) MPa Young’s modulus and the soft substrate with (~0.046 ± 0.020) MPa Young’s modulus. The parameters characterizing the substrate roughness and stiffness were in our previous data.^17–19^

### Cell culture

*For* chondrocytes, the cells were isolated from the knee joint of newborn C57BL mice. The isolation method was followed by the maturation protocol.^45,46^ Briefly, the collected knee joint without epidermis was trypsinised at a concentration of 0.25% for 20–30 min. After removal of trypsin, the lysate with 0.5% collagenase type II was digested for 3–4 h. Then, the chondrocyte suspension was mixed 1:1 with 10% foetal bovine serum, high-glucose Dulbecco’s modified Eagle’s medium (FBS-DMEM, Thermo Fisher Scientific, Waltham, MA) with 0.1 mmol·L^−1^ non-essential amino acids, 4 mmol·L^−1^ L-glutamine, and 1% penicillin streptomycin solution. The mixture was centrifuged at 1 000 r·min^−1^ for 5 min. The collected chondrocytes were then resuspended in fresh 10% FBS DMEM. After cell counting, primary chondrocytes were seeded onto substrates with different stiffnesses at 37 °C in a humidified atmosphere of 5% CO_2_.

*For* osteoblasts, the cells were isolated from the skull of newborn C57BL mice. The isolation method was followed by the maturation protocol.^47^ Briefly, the skulls were first cut into small pieces in aseptic phosphate-buffered saline (PBS, 1×). Next, the tissue fragments were digested in MEM alpha basic (α-MEM, Thermo Fisher Scientific) with 0.5% collagenase type I overnight. Then, the primary osteoblasts were collected by centrifugation at 1 000 r·min^−1^ for 5 min. The cells were resuspended in fresh 10% FBS α-MEM. After cell counting, primary osteoblasts were seeded onto substrates with different stiffnesses at 37 °C in a humidified atmosphere of 5% CO_2_.

*For* osteoclasts, the cells originated from bone marrow macrophages (BMMs) in the femurs of one-month-old C57BL mice. The isolation method was followed by the maturation protocol.^48^ Briefly, we first collected BMMs in aseptic conditions and resuspended them in 10% FBS αMEM with macrophage colony stimulating factor (MCSF, SRP3221, Sigma, St. Louis, MO) at a concentration of 40 ng·mL^−1^ for 24 h. The cells were then transferred onto substrates with different stiffnesses and cultured in 10% FBS α-MEM with 20 ng·mL^−1^ M-CSF and 20 ng/ml Receptor Activator of NF-kB ligand (RANKL, R0525, Sigma). We induced BMMs by changing half media every day. After approximately 4 days of induction, large fused osteoclasts were formed.

*For* osteocytes, we used the cell line MLO-Y4, which was purchased from the University of Texas. The culture method was described previously.^19^ MLO-Y4 cells were maintained in 10% FBS DMEM containing 4.5 g·L^−1^ glucose, 0.1 mM nonessential amino acids, 4 mmol·L^−1^ L-glutamine and 1% penicillin/streptomycin (V/V). After cell counting, the MLO-Y4 cells were seeded onto substrates with different stiffnesses.

*For bone marrow-derived stromal* cells (BMSCs), the cells were collected from the bone marrow in the femurs of one-month-old C57BL mice. The cell culture method was followed by the maturation protocol. Briefly, the femurs were collected with ophthalmic scissors, then washed in PBS with 5% penicillin/streptomycin and transferred to PBS with 1% penicillin/streptomycin to avoid contamination. The two head of the femurs were cut, and the all bone marrow cells were collected with α-MEM. The cells were collected in 15 mL tubes and centrifuged at 1000 r·min^−1^ for 5 min. The cells were collected and resuspended in 10% FBS α-MEM. Finally, the cell suspension was transferred onto the Petri dish and cultured at 37 °C in a humidified atmosphere of 5% CO_2_. BMSCs would attach the bottom of the Petri dish for approximately 3–5 days. After that, fresh medium was changed, and the cells were collected for PDMS substrate seeding.

### Cell morphology test

After 72 h seeding onto PDMS substrates with different stiffnesses, the cells (chondrocytes, osteoblasts osteocytes and BMSCs) were imaged by phase contrast microscopy (IX2-1LL100, Tokyo, Japan), and the data about quantification of cell spreading areas were calculated by its connected software, Image-Pro Plus 6.0 (IPP 6.0). The statistical data are presented in the form of histograms.

### Immunofluorescence

Cells were washed three times with ×1 PBS and then fixed with 4% paraformaldehyde (PFA) for 12 min. After being penetrated by Triton X-100 (0.25%–0.5%) for 5 min, the cells were blocked by bovine serum albumin (BSA, 5%) for 2 h. Cells were incubated with antibodies, e.g., phalloidin (FITC, A12379, Thermo) and vinculin (ab196579, Abcam, Cambridge, UK) antibodies. Then, 2-(4-amidinophenyl)-6-indolecarbamidine dihydrochloride (Dapi, D9542, Sigma, St. Louis, MO) was applied to stain the nuclei for 10 min at 10 μg·mL^−1^. The immunofluorescence images of cell samples were observed through a confocal laser scanning microscope (FV3000, Olympus, Tokyo, Japan and A1R MP+, Nikon, Tokyo, Japan, and parameter: 40, Nikon Microsystems original image: 1 024 × 1 024). The images presented in the figures are representative of a projection of all slices based on at least three independent experiments (*n* = 3). For the quantification of immunofluorescence intensity, we used the connected software in FV3000, Olympus and A1R MP+, Nikon.

### Western blotting

Cell lysates were mixed with 1:1 loading buffer and boiled for 5 min after being quantified using a BCA kit (P0010S, Beyotime, Shanghai, CHINA). The protein samples were separated by 10% sodium dodecyl sulphate polyacrylamide gel electrophoresis (SDS-PAGE) and then transferred to a PVDF membrane at 200 mA for 2 h at room temperature (RT). The PVDF membranes with protein blots were blocked in 5% nonfat milk for ~2 h and then washed away with TBST buffer three times. The PVDF membranes with protein blots were then incubated with vinculin antibody (ab129002, Abcam) and β-actin (sc-47778, Santa Cruz Biotech, Delaware Avenue, CA) for 2 h at 37 °C (or overnight at 4 °C). The secondary antibodies were mouse anti-rabbit IgG-HRP (sc-2357, Santa Cruz) and m-IgGκ BP-HRP (sc-516102, Santa Cruz). Signals from blots were obtained using a Santa Cruz Western Blotting Luminol Reagent Kit (sc-2048).

### Statistical analysis

The analysed data are shown as the mean ± SEM. Data were based on at least three independent experiments (*n* = 3). Statistical analysis was performed by one-way analysis of variance (ANOVA) to determine the differences among groups. Post hoc analysis utilized Fisher’s protected least significant differences (PLSD). In each analysis, the critical significance level was set at *P* < 0.05.

## Data Availability

The authors declare that the data supporting the findings of this study are available within the article.
